# Pharmacovigilance on olanzapine

**DOI:** 10.4103/0253-7613.68438

**Published:** 2010-08

**Authors:** Anand Lingeswaran, Harsha Shetty, Kigshuk Lahon, Amit Paramel, Gyaneshwar Sharma

**Affiliations:** Department of Psychiatry, Indira Gandhi Medical College and Research Institute, Kathirkamam, Vazhudavur Road, Pondicherry - 605 009, India. E-mail: anafonix@gmail.com; 1 Department of Pharmacology, Mahatma Gandhi Medical College & Research Institute, Pillayarkuppam, Pondy-Cuddalore Road, Pondicherry-607402, India; 2 Department of Psychiatry, Mahatma Gandhi Medical College & Research Institute, Pillayarkuppam, Pondy-Cuddalore Road, Pondicherry-607402, India

Sir,

Pharmacovigilance, the science and activities relating to the detection, assessment, understanding and prevention of adverse effects or any other drug-related problems, is highly essential in India, where there is a lack of adequate efficacy and safety-related data, limited efforts made on adverse drug reaction (ADR) monitoring by the manufacturers, lack of ADR data for traditional medicines and absence of structural and policy changes within the drug regulatory authorities.

Olanzapine has adverse effects such as dystonic reactions, akathisia, tardive dyskinesia, tremors, hyperprolactinemia, weight gain, neuroleptic malignant syndrome and metabolic syndrome. In a busy tertiary care hospital setting, it is not unusual for psychiatrists to prescribe olanzapine without checking for the presence of multiple medical illnesses comorbid with major mental illness. Olanzapine can cause life-threatening adverse drug to drug interactions. In this respect, ADR reporting within the hospital is highly imperative.

We designed a retrospective chart review to study the method of ADR monitoring in olanzapine-treated patients. The study was conducted at the Department of Pharmacology and Psychiatry at Mahatma Gandhi Medical College and Research Institute, Sri Balaji Vidyapeeth, Pondicherry, India, from 1^st^ January 2006 to 31^st^ December 2006. All cases that had been started on oral olanzapine treatment and followed-up for more than 8 weeks were recruited for the study. Using a semi-structured proforma, the personal and clinical data such as age, sex, body weight in kilograms and clinical diagnoses (ICD 10), name of medication, dosage and its titration in the 1-year period were recorded. Documentation of adverse effects and laboratory investigation details were also recorded. The Naranjo’s scale (WHO) was used for studying the causal relation between olanzapine and the observed adverse event. Statsdirect, statistical software, Version 2.7.2 was used for analysis.

The sample comprised of 71 cases, all with the diagnosis of schizophrenia of various subtypes (ICD-10), 28 (39.4%) cases had documented at least one ADR. Mean age of the patients was 28.3 years and mean duration of schizophrenic illness was 2.2 years.

Of the 71 cases, 13 cases (18.3%) (six males and seven females) had documented weight gain. The mean age of all 13 cases was 29.84 years (SD, 11.95). The mean dose of olanzapine in the 13 cases was 6.7 mg/day, lower when compared with evidence from clinical trials that show 10–20 mg/day as the most effective dose. The overall weight difference (mean of differences -4.53; t -4.91; *P* < 0.001) of the 13 cases was highly significant statistically and clinically [Figure 1]. The ladder plot depicts the individual sex differences of weight gain [Figure 2a and b]. The Shapiro-Wilk W test showed no evidence of non-normality (W = 0.89; *P* = 0.123), which makes the findings of this study generalizable.

**Figure 1:**
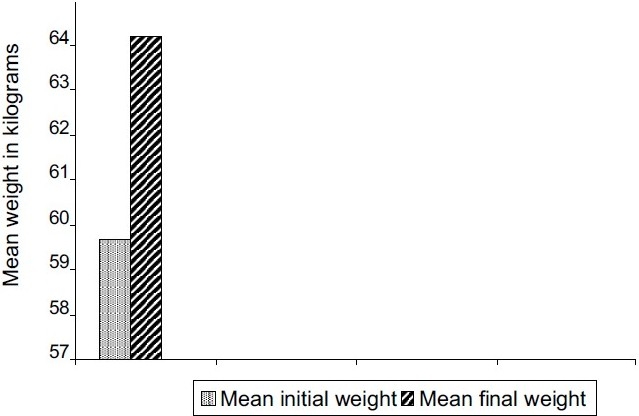
Mean weight at baseline and at 8 weeks

**Figure 2a:**
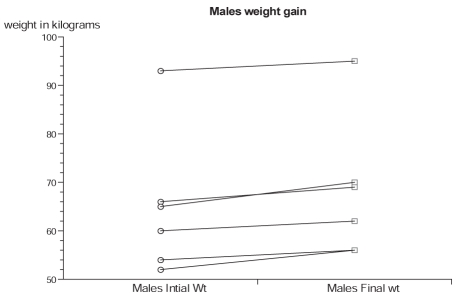
Weight gain in males over 8 weeks

**Figure 2b:**
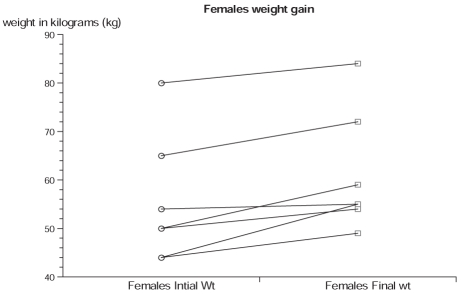
Weight gain in females over 8 weeks

Five cases (7.0%) developed tremors only and five (7.0%) developed increased appetite. Of the 71 cases, three (4.2%) cases had developed laboratory-determined hyperglycaemia. On the Naranjo’s scale, the results were weight gain (score 2–5; possible to probable), tremors (6–7; probable), increased appetite (2–3; possible) and hyperglycaemia (6–7; probable).

As per the Australian Adverse Drug Research Council report[1] on suspected adverse reactions with olanzapine, the two most frequent adverse reactions in clinical trials were somnolence and weight gain. Our results were different probably because of the long duration of schizophrenia itself, making the patient vulnerable to extrapyramidal side-effects (EPS) and weight gain. However, weight gain is a commonly reported ADR due to olanzapine. There is enough evidence to state that olanzapine is 10-times more likely to produce weight gain, seen in 8 weeks, than placebo in the treatment of schizophrenia.[2,3] Surprisingly, the low mean dose of 6.7 mg/day in our study had produced similar weight gain in contrast to studies that report that a mean daily dose of 15 mg (SD, 2.5) of olanzapine increased the body weight by 11.8 kg in 1 year.[4]

The low incidence of EPS with olanzapine can be explained on the basis of its greater affinity for 5-HT2 receptors than for D2 receptors hence causing fewer EPS in contrast with classical antipsychotics (e.g., haloperidol). Olanzapine is well known to cause weight gain and presumably increased appetite as an initial symptom in the course of treatment. A large number of case reports have demonstrated the link between hyperglycaemia during olanzapine treatment.[5]

The differences in findings observed in other countries and those reported in this study highlight the need for developing our own local and national-level ADR database. India seems to rate below 1% in ADR reporting, as against the world rate of 5%. A national regulatory body should be established to implement the system of reporting adverse events of drugs introduced in the Indian market by pharmaceutical companies. Similarly, ADR audits will prove useful in hospitals too. Finally, the government has to play an effective regulatory role in ensuring the availability of safe medicines to the consumers.

## References

[1] Casey DE, Haupt DW, Newcomer JW, Henderson DC, Sernyak MJ, Davidson M (2004). Antipsychotic-induced weight gain and metabolic abnormalities: Implications for increased mortality in patients with schizophrenia. J Clin Psychiatry.

[2] Kinon BJ, Kaiser CJ, Ahmed S (2005). Association between early and rapid weight gain and change in weight over one year of olanzapine therapy in patients with schizophrenia and related disorders. J Clin Psychopharmacol.

[3] Beasley CM, Tollefson GD, Tran PV (1997). Safety of olanzapine. J Clin Psychiatry.

[4] Azriel Mira S (2002). Uncontrolled hyperglycaemia with ketosis associated with olanzapine therapy. Rev Clin Esp.

[5] Torrey EF, Swalwell CI (2003). Fatal olanzapine-induced ketoacidosis. Am J Psychiatry.

